# Development and Validation of an Artificial Intelligence-Based Image Classification Method for Pathological Diagnosis in Patients With Extramammary Paget’s Disease

**DOI:** 10.3389/fonc.2021.810909

**Published:** 2022-01-18

**Authors:** Hao Wu, Huyan Chen, Xuchao Wang, Liheng Yu, Zekuan Yu, Zhijie Shi, Jinhua Xu, Biqin Dong, Shujin Zhu

**Affiliations:** ^1^ Department of Dermatology, Huashan Hospital, Fudan University, Shanghai, China; ^2^ Academy for Engineering and Technology, Fudan University, Shanghai, China; ^3^ Shanghai Medical School, Fudan University, Shanghai, China; ^4^ Nanjing University of Posts and Telecommunications, Nanjing, China

**Keywords:** extramammary Paget’s diseases, pathological diagnosis, computer-aided diagnostic solution, artificial intelligence, deep learning

## Abstract

Extramammary Paget’s disease (EMPD) is a rare, malignant cutaneous adenocarcinoma with a high recurrence rate after surgical resection. Early diagnosis of EMPD is critical as 15%–40% of cases progress into an invasive form and resulting in a dismal prognosis. However, EMPD can be a diagnostic challenge to pathologists, especially in the grassroots hospital, because of its low incidence and nonspecific clinical presentation. Although AI-enabled computer-aided diagnosis solutions have been extensively used in dermatological pathological image analysis to diagnose common skin cancers such as melanoma and basal cell carcinoma, these techniques have yet been applied to diagnose EMPD. Here, we developed and verified a deep learning method with five different deep convolutional neural networks, named ResNet34, ResNet50, MobileNetV2, GoogLeNet, and VGG16, in Asian EMPD pathological image screening to distinguish between Paget’s and normal cells. We further demonstrated that the results of the proposed method are quantitative, fast, and repeatable by a retrospective single-center study. The ResNet34 model achieved the best performance with an accuracy of 95.522% in pathological images collected at a magnification of ×40. We envision this method can potentially empower grassroots pathologists’ efficiency and accuracy as well as to ultimately provide better patient care.

## 1 Introduction

Extramammary Paget’s diseases (EMPD) is a rare cutaneous malignancy characterized histopathologically by epidermal adenocarcinoma cells (Paget’s cells) (1, 2). EMPD primarily localizes to the penis, scrotum, vulva, perianal, and axillary regions (3). The incidence of EMPD ranges from 0.1 to 2.4 patients per million person-years. It mainly occurs in elderly people aged 60 to 80. The prevalence of EMPD in Asians (10 cases per million) is higher than Westerners (0.9 cases per million) (3). Typically, the course of EMPD is indolent. The skin lesions are manifested as infiltrating erythema, which may be accompanied by erosions. Repetitive excoriation may modify the appearance of skin lesions leading to misdiagnoses, such as fungal infection eczema, or other diseases, resulting in delay of treatment. About 15% to 40% of all EMPD cases progress into an invasive form, which may cause poor prognoses (4–6). Therefore, the early diagnosis of EMPD is very important.

**Figure 1:**
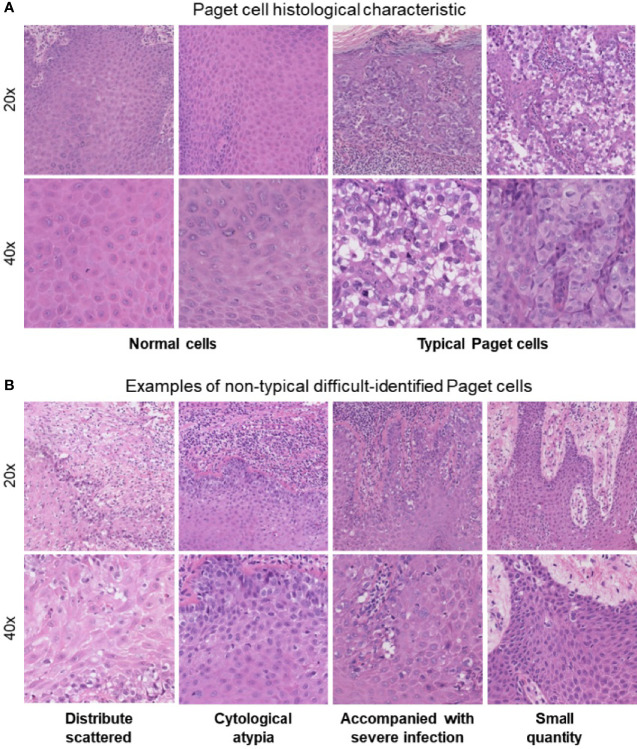
**(A)** Paget’s cell histological characteristics: abundant pale cytoplasm and large nuclei with a prominent, vesicular nucleus with H&E stain when compared with normal cells. **(B)** Atypical difficultly identified Paget’s cells.

At present, a punch biopsy is necessary to make a definite diagnosis of EMPD. The morphological characteristics of Paget’s cells can be confirmed through the use of hematoxylin and eosin (H&E) staining (7). Traditional assessment of stained histology slides mainly relies on human visual observation, which not only leads to inefficient pathology workflow but also causes misdiagnosis or missed diagnosis due to subjective factors. EMPD can be a diagnostic challenge to pathologists because of its low incidence and nonspecific clinical presentation, especially in the grass-roots hospital. Typical Paget’s cells have abundant pale cytoplasm and large nuclei with a vesicular, prominent nucleus with H&E stain when compared to normal cells (**Figure 1A**). However, in atypical cases shown in **Figure 1B**, there are more challenges for pathologists to identify EMPD from other diseases. First of all, Paget’s cells usually show atypical in the early stage. Secondly, Paget’s cells usually distribute scattered, and the quantity of cells might be very small. Thirdly, in cases where the lesion is accompanied by severe infection, Paget’s cells usually mix with a large amount of inflammatory cells. All these situations may cause the pathologists to fail to distinguish tumor cells. Even if the slice is stained by immunohistochemistry, there will still be a risk of missed diagnosis or misdiagnosis (8). Missed diagnosis or misdiagnosis may result in expanding the surgical resection range, and reducing the quality of patients’ life. Based on these characteristics, the pathological diagnosis of EMPD usually requires experienced pathologists to make a clear diagnosis. As the incidence of EMPD is increasing yearly, the workload of pathologists has significantly increased. Developing an application in the diagnosis of EMPD to help pathologists improve work efficiency as well as avoiding missed diagnoses is urgent.

Nowadays, AI-enabled computer-aided pathological diagnosis solutions have been used extensively in clinical practice, which can provide pathologists with stable and reliable digital workflows. The evolution of these diagnostic decision support tools can improve the accuracy and efficiency of pathologists and provide better medical services to patients (9). Thomas et al. found that some of the most common skin cancer diagnoses, such as basal cell carcinoma (BCC), squamous cell carcinoma (SCC), and intraepidermal carcinoma (IEC) are amenable to deep learning methods (10). Li et al. proposed a deep learning-based pathology diagnosis system for melanoma whole slide imaging (WSI) classification and generated a multicenter WSI database for model training, which could assist the pathological diagnosis of melanoma diseases (11). Xie et al. collected 2,241 digital whole-slide images from 1,321 patients and constructed a multicenter dataset for training both ResNet50 and Vgg19 to test performance with the classification of melanoma and nevi. The study achieved high accuracy in distinguishing melanoma from nevi with a sensitivity of 0.92 and a specificity of 0.94 (12). Heckler et al. trained a ResNet50 network by 595 pathological images to compare the performance of pathologists in classifying melanoma and nevi. The diagnostic discordance was 18% for melanoma and 20% for nevi (13). All these previous studies focused on melanoma, SCC, or BCC which comprise 98% of all skin cancers.

However, dermatological pathological image analysis techniques are only applied to diagnose common skin cancers such as BCC, SCC, and melanoma (13, 14). Other skin cancers, including cutaneous lymphoma, EMPD, Merkel cell carcinoma, and Kaposi’s sarcoma are ignored by most algorithms mainly due to the long period required for pathology image collection and doubts about the ability of deep learning on more complicated histopathology. Therefore, this paper was specifically designed to reveal the recognition ability of EMPD using convolutional networks and evaluate its ability to produce explainable and interpretable predictions. The main contributions of this paper can be summarized as follows: (1) to the best of our knowledge, we for the first time used the deep learning method in EMPD pathological image rapid screening to distinguish between Paget’s cells and normal cells, which is helpful for assisting doctors in diagnosis; (2) we demonstrated the classification of skin diseases using ResNet34, ResNet50, MobileNetV2, GoogLeNet, and VGG16 by transfer learning strategy, using only pixels and disease labels as inputs. Extensive experiments simulating the clinical diagnosis process are conducted to evaluate the effectiveness; (3) we built a single-center database of 584 WSIs including 341 cases from 286 EMPD patients and 243 normal skin images. This data-driven approach can overcome the challenge that arises from morphological diversity in histopathological images.

## 2 Materials and Methods

### 2.1 Sample Population and Dataset

In this study, we collected 584 H&E-stained whole-slide skin histopathology images at different magnifications, including 341 abnormal skin images and 243 normal skin images from the Dermatological Pathology Department of Huashan Hospital, Fudan University. Patients’ clinical characteristics are shown in **Table 1**. These slides were scanned with size of 1,920 × 1,088 pixels at two different magnifications, ×20 (0.5 µm/pixel) and ×40 (0.275 µm/pixel), by a digital slice scanning system (NanoZoomer 2.0-RS, Hamamatsu Photonics, Hamamatsu, Japan). The original pathological images were cropped manually by experienced dermatology pathologists. This study was approved by the ethics committees of Huashan Institutional Review Board (HIRB) (Approval Number: 2021-901).

**Table 1:** EMPD patients’ clinical characteristics from 2009 to 2021.

EMPD patients clinical characteristics		Male	Female	Total	p-value
		*N* = 241	*N* = 45	*N* = 286	
***Age (year)***	***Median***	69	67	69	0.070
	***Range***	45–92	34–90	34–92
***Site of lesion***	***Vulva (%)***	206 (85.48)	32 (71.11)	238 (83.22)	0.046
	***Trunk (%)***	35 (14.53)	13 (28.89)	48 (16.78)
***Clinical diagnose accordance rate***	***Y (%)***	184 (76.35)	16 (35.56)	200 (69.93)	0.001
	***N (%)***	57 (23.65)	29 (64.44)	86 (30.07)

### 2.2 Preprocessing

We randomly crop the collected skin pathology images into different sizes and aspect ratios and scale the cropped image to a size of 224 × 224 pixels. We then randomly flip images horizontally with a probability of 0.5, convert images to tensors, and perform pixel normalization processing on the image. The value of each pixel in the image is divided by 255 to convert it to between [0, 1].

### 2.3 Experiment Procedure

**Figure 2:**
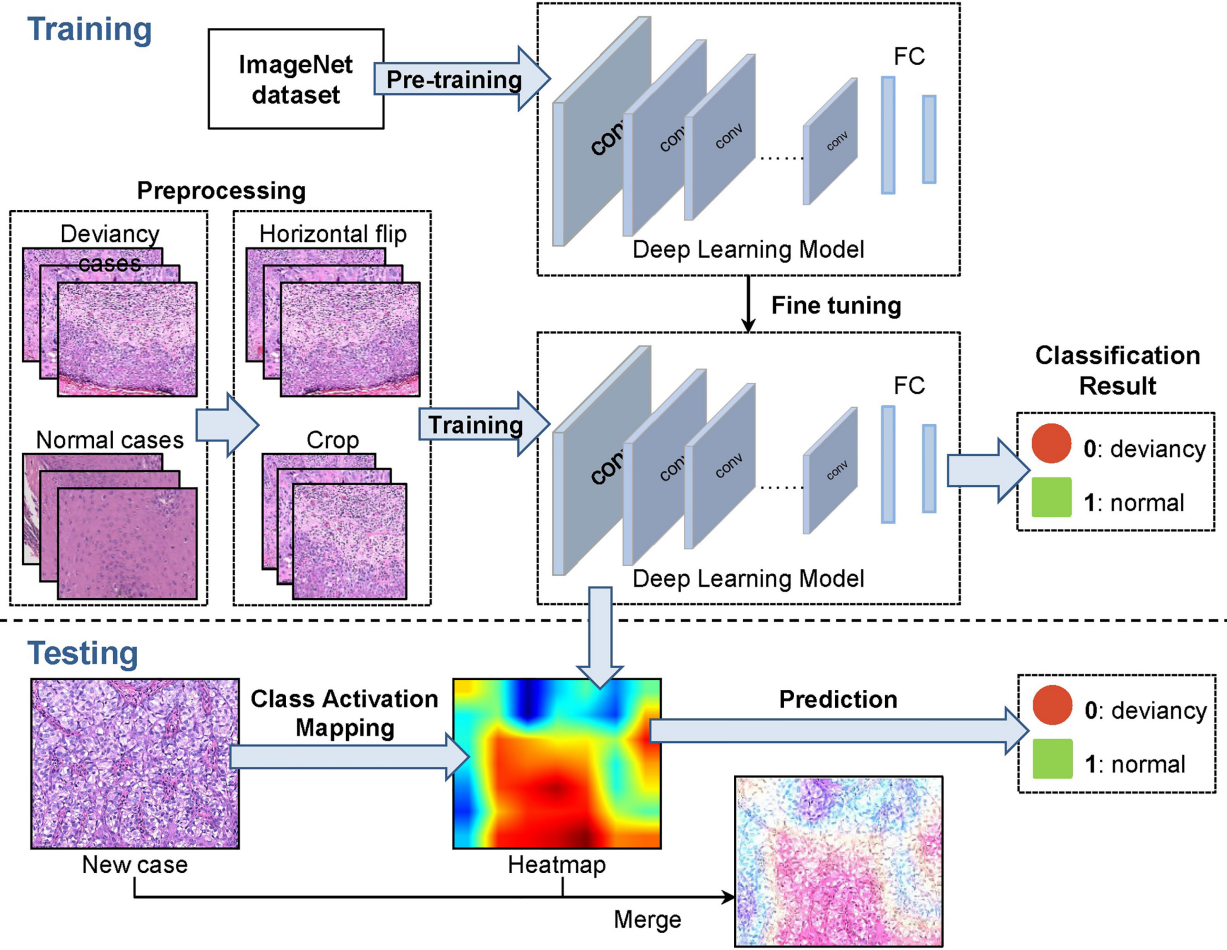
A representative methodological pipeline of the EMPD computer-aided diagnosis system.

The methodological pipeline of the EMPD computer-aided diagnosis system is shown in **Figure 2**. To train the deep network model, the collected 584 skin images are randomly divided into training set, validation set, and test set with the ratio of 6:2:2. After this division, 375 images are included in the training set, 109 images are included in the validation set, and the rest 100 images are then regarded as the testing set. As different deep network models were designed, or optimized for some specific tasks, we only exploited the widely used and accepted deep neural networks which are VGG16, GoogLeNet, ResNet34, ResNet50, and MobileNetV2. To build the EMPD pathological image classifiers, the 375 skin image samples with corresponding labels are fed to different deep network models to train the network. In the training step, all images from the validation set are also involved to avoid overfitting and tune the network hyperparameters during the model configuration. Finally, the testing images are then fed to the built deep networks model to evaluate the classification performance by giving its class prediction.

### 2.4 Convolutional Neural Networks

Deep convolutional neural networks (CNN) have made great progress in various computer vision tasks including image classification, object detection, and semantic segmentation. The basic convolutional neural network mainly consists of an input layer, a convolutional layer, an activation layer, a pooling layer, and a fully connected layer. The convolutional operation is used to extract image features. The pooling operation is used to reduce the dimension and improve the calculation speed. The activation function is used to introduce nonlinear factors to improve the expressiveness of the model and the fully connected layer can connect the output features of all layers and send the output values to the classifier for classification.

#### 2.4.1 VGGNet

In 2014, VGG was proposed by the prestigious Visual Geometry Group (VGG) at the Oxford University and won the first place in the Localization Task and second place in the Classification Task of the ImageNet competition that year. VGGNet (15) is an improved version of classical AlexNet, and the whole model uses 3 × 3 convolution kernels instead of 5 × 5 convolution kernels, and three 3 × 3 convolution kernels instead of 7 × 7 convolution kernels, which reduces the number of parameters and is easy to be trained. Moreover, the VGGNet has deeper architectures than the classical convolutional network which bring better capacity in extracting image features.

In this work, the VGGNet with 16 weight layers named VGG16 is used, which can be divided into feature extraction network structure and classification network structure. In extracting features, it is mainly realized by convolution operation and maximum pooling operation. All convolutional layers have the same configuration. In the classification network, there are three fully connected layers, and the classification result is obtained through Softmax processing. All hidden layers are followed by the ReLu nonlinear activation function.

#### 2.4.2 GoogLeNet

In 2014, the Google team proposed the GoogLeNet model which performed better than the state of the art methods in the Classification Task of the ImageNet competition that year. The Inception structure is introduced in GoogLeNet (16), which can fuse feature information of different scales. GoogLeNet model uses 1 × 1 convolution to reduce dimensionality, adds two auxiliary classifiers to help training, discards the fully connected layer instead of using the fully connected layer, Which greatly reduces the model effectively.

#### 2.4.3 ResNet

In 2015, ResNet was presented by Microsoft Labs, which won the first place in the Classification Task, Target Detection, and Image Segmentation Task of the ImageNet competition that year. Generally, deeper network architectures bring better ability in feature extraction. However, the increase of network depth also leads to the problem of gradient disappearance and explosion during the training stage. The ResNet (17) introduces the residual module and batch normalization to avoid the gradient disappearance and explosion problem and to accelerate network training.

**Figure 3:**
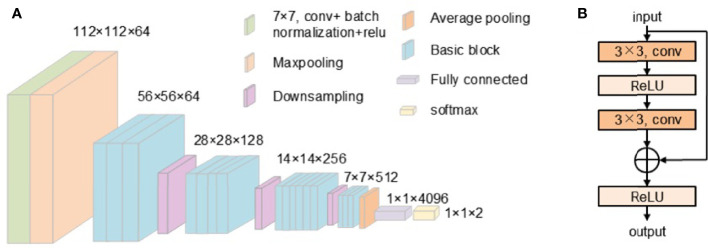
**(A)** The structure of ResNet34. **(B)** The structure of the basic block.

This work uses ResNet34 and ResNet50 to classify skin pathology images. ResNet34 uses basic block, which is mainly composed of two 3 × 3 convolutions (**Figure 2**). ResNet50 uses bottleneck. The bottleneck module uses 1 × 1, 3 × 3, and 1 × 1 convolution, which can reduce the number of network parameters. The network structure of ResNet34 used in our work is shown in **Figure 3**.

#### 2.4.4 MobileNet

MobileNet, proposed by the Google team in 2017, focuses on lightweight CNN networks in mobile or embedded devices. Compared with traditional convolutional neural networks, it greatly reduces model parameters and operations with a small reduction in accuracy. MobileNet is mainly composed of depth-wise convolution and point-wise convolution (18), which is a deep separable convolution. In conventional convolution, the convolution kernel acts on all input channels, while in depth-wise convolution, a different convolution kernel is used for each input channel to perform the convolution operation independently. The point-wise convolution uses 1 × 1 convolution kernel, and combining the obtained features and using the depth separable convolution can greatly reduce the amount of calculation and the number of model parameters.

The MobileNetV2 model used in this work was proposed by the Google team in 2018 and is more accurate and has a smaller model compared with the MobileNetV1 network. It has inverted residuals and a linear bottleneck. The inverted residuals block is composed of 1 × 1 convolution for dimension enhancement, 3 × 3 depth-wise convolution, and 1 × 1 point-wise convolution for dimension reduction. Linear bottleneck does not perform ReLu function processing after the last point-wise convolution and outputs directly.

### 2.5 Loss Function

In our work, the cross-entropy loss function (19) is employed for all deep models. The cross-entropy loss is computed as follows:


$$\text{loss}(y,p)=-\frac{1}{N}\sum_{i=1}^{N}\sum_{c=1}^{C}y_{i,c}\cdot \log(p_{i,c})$$

where *y_i,c_* is the one-hot encoding format of ground truth labels, *p_i,c_* is the matrix of predicted values for pixels in each class, where the indices, *c* and *i*, iterate over all classes and pixels, respectively. Cross-entropy loss is based on minimizing pixel-wise error, where for class imbalanced cases, it may lead to an over-representation for dominant class samples in the loss, resulting in the poor representation or weak contribution for minority samples.

### 2.6 Transfer Learning

Generally, to solve a complex task or to build a well-performed deep network model, a vast amount of samples and labeled data is required. However, in the cases with insufficient amount of training data, the trained model may fail to extract good feature and suffer from the significant loss in performance. The idea of transfer learning is to utilize the knowledge acquired from previously learned task to solve related ones effectively and efficiently. Through transfer learning, the knowledge trained on the source task can be transferred to the application of the target task (20). Thus, the transfer learning method can train a well-performed model easily even with a small amount of dataset.

In the experiment of this work, the transfer learning strategy is adopted and the initial weights of deep neural models (except for fully connected layer) are inherited from its pretrained models trained by ImageNet. The last fully connected layer of the network is modified to meet our classification for each network models.

### 2.7 Evaluation Index

#### 2.7.1 Confusion Matrix

The confusion matrix can visualize the performance of the algorithm and gives the insight of what your classification model is getting right and what types of errors it is making (21). The calculation of confusion matrix is shown in **Table 2**. It is necessary to separately count the number of observations of the correct category and the number of observations of the predicted error category in the model.

**Table 2:** The confusion matrix.

Confusion matrix	Predicted class	
	Positive	Negative
Actual class		
Positive	TP	FN
Negative	FP	TN

True positive (TP) is a positive example and the model prediction is a positive example. True negative (TN) is a negative example and the model prediction is a negative example. False positive (FP) is a negative example, but the model prediction is a positive example. False negative (FN) is a positive example, but the model prediction is a negative example.

#### 2.7.2 Index Calculation

##### 2.7.2.1 Accuracy

The accuracy rate is the proportion of all the correct judgments in the model among all the predicted values. The formula is as follows:


$$\text{acc}=\frac{\text{TP}+\text{TN}}{\text{TP}+\text{TN}+\text{FP}+\text{FN}}$$

##### 2.7.2.2 Precision

Precision gives an indication of the ability not to predict negative samples as positive samples. The precision rate is the proportion of the predictions that are correct in the result of the model prediction as a positive example. The formula is as follows:


$$\text{precision}=\frac{\text{TP}}{\text{TP}+\text{FP}}$$

##### 2.7.2.3 Recall

The recall rate is the proportion of positive examples correctly predicted by the model among all the positive examples in the dataset. The recall rate can reflect the ability of the model to predict positive samples. The formula is as follows:


$$\text{recall}=\frac{\text{TP}}{\text{TP}+\text{FN}}$$

##### 2.7.2.4 F1-Score

The F1-score combines the results of precision and recall. The value of the F1-score ranges from 0 to 1: 1 represents the best output of the model and 0 represents the worst output of the model. The F1-score reflects the stability of the model, and the higher the F1-score is, the more robust the model is. The formula is as follows:


$$\text{F}1=\frac{2\text{TP}}{2\text{TP}+\text{FP}+\text{FN}}$$

##### 2.7.2.5 ROC Curve

The ROC curve is the receiver operating characteristic curve, and there are two main indicators in the ROC curve: false-positive rate (FPR) and true-positive rate (TPR). The FPR indicates the degree of response falsely reported by the model, and the TPR indicates the corresponding degree of coverage predicted by the model. The higher the TPR and the lower the FPR, the more effective the model is. The formula is as follows:

##### 2.7.2.6 Sensitivity

Sensitivity measures the percentage of the positive sample that was predicted to be positive. It reflects the ability of the model to correctly predict positive samples. The formula is as follows:

##### 2.7.2.7 Specificity

Specificity measures percentage of the negative sample that was correctly predicted to be negative. It reflects the ability of the model to correctly predict negative samples. The formula is as follows:

##### 2.7.2.8 Auc-score

Auc is the area under the ROC curve. The larger the Auc-score values, the higher the accuracy of the classifier is.

## 3 Results and Discussion

**Figure 4:**
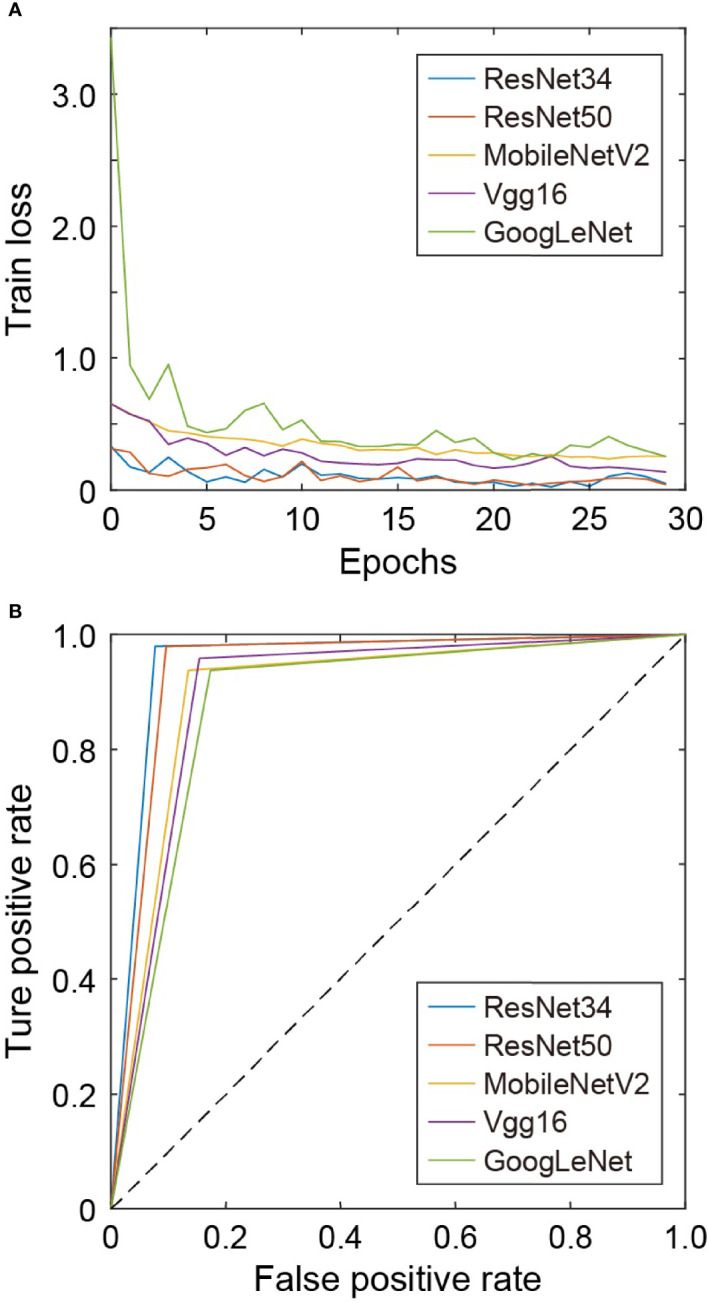
**(A)** Training loss under different networks. **(B)** ROC curves under different networks.

For all network models in this work, the cross-entropy loss function and Adam optimizer are used. The initial learning rate is 0.0001, and the training iterations is 30 epochs. The training loss of each network model is shown in **Figure 4A**. For VGG16, ResNet34, and ResNet50, the loss fluctuates greatly during the training process and the convergence is slow, while for GoogLeNet, the loss decreases rapidly at the beginning and then changes slowly afterwards. The loss of MobileNetV2 decreases slowly with small fluctuations, and its convergence state is relatively better. **Figure 4B** shows the ROC curves under different network models for comparison. The test set has the ROC curve closest to the upper left corner on ResNet34, followed by that on ResNet50.

**Table 3** gives the time taken to predict 100 skin images under different network models. MobileNetV2 takes relatively the least time to predict due to its lightweight structure, while VGG16 takes relatively the longest time due to its deeper network structure with more parameters and heavier calculations.

**Table 3:** The time of testing 100 skin images under different network models.

Model	Time(s)
VGG16	24.5982
GoogLeNet	12.3033
**ResNet34**	13.6707
ResNet50	17.6632
MobileNetV2	**11.9645**

The shortest time is highlighted in bold.

The evaluation indicators use accuracy, precision, recall, F1-score, Auc-score, sensitivity, and specificity. In total, 100 skin pathological images were tested. The accuracy and Auc-score under different network models are shown in **Table 4**. In the ResNet34 model, the prediction accuracy and Auc-score are the highest, reaching 0.9500 and 0.9511. Among the 100 testing images, 95 skin pathology images can be correctly distinguished. In contrast, GoogLeNet has the lowest prediction accuracy and the lowest Auc-score.

**Table 4:** Accuracy and Auc-score under different network models.

Model	Accuracy	Auc-score
VGG16	0.9000	0.9022
GoogLeNet	0.8800	0.8822
**ResNet34**	**0.9500**	**0.9511**
ResNet50	0.9400	0.9415
MobileNetV2	0.9000	0.9014

The highest accuracy is highlighted in bold.

**Table 5** shows the precision, recall, F1-score, sensitivity, and specificity predicted on the normal skin image and the deviancy skin image under different network models. It can be seen that in each model, the precision of deviancy skin images is higher than that of normal images.

**Table 5:** The accuracy, recall, and F1-score predicted on the normal skin image and the abnormal skin image under different network models.

Model	Skin type	Precision	Recall	F1-score	Sensitivity	Specificity
VGG16	Deviancy	0.9565	0.8462	0.9000	0.8654	0.9375
	Normal	0.8519	0.9375	0.9000	0.9375	0.8654
GoogLeNet	Deviancy	0.9348	0.8269	0.8776	0.8269	0.9375
	Normal	0.8333	0.9375	0.8824	0.9375	0.8333
ResNet34	Deviancy	**0.9796**	**0.9231**	**0.9505**	**0.9231**	**0.9792**
	Normal	**0.9216**	**0.9792**	**0.9495**	**0.9792**	**0.9231**
ResNet50	Deviancy	0.9792	0.9038	0.9400	0.9038	0.9792
	Normal	0.9038	0.9792	0.9400	0.9792	0.9038
MobileNetV2	Deviancy	0.9375	0.8654	0.9000	0.8654	0.9375
	Normal	0.8654	0.9375	0.9000	0.9375	0.8654

The optimal results are highlighted in bold.

In the ResNet34 network, when predicting normal skin images and deviancy skin images, the prediction precision, recall, F1-score, sensitivity, and specificity are the highest. Therefore, compared with the other four network structures, ResNet34 is more suitable for the classification of the giving skin pathology dataset with high stability and predictive accuracy.

Taking into account the effect of the magnification of the skin pathology image on the classification results, when the magnification is larger, the tissue characteristics will be more obvious, which will have a certain impact on the recognition of the lesion. In the work of this paper, the testing data are predicted according to different magnifications. The accuracy and Auc-score of ResNet34 model at ×20 and ×40 magnifications of the skin pathology image are shown in **Table 6**. It can be seen the accuracy is slightly higher at ×40 than that at ×20, but the difference in Auc-score is not significant.

**Table 6:** The accuracy and Auc-score of ResNet34 model at different magnifications.

Magnifications	Accuracy	Auc-score
**×20**	0.9355	0.9524
**×40**	0.9552	0.9524

**Figure 5:**
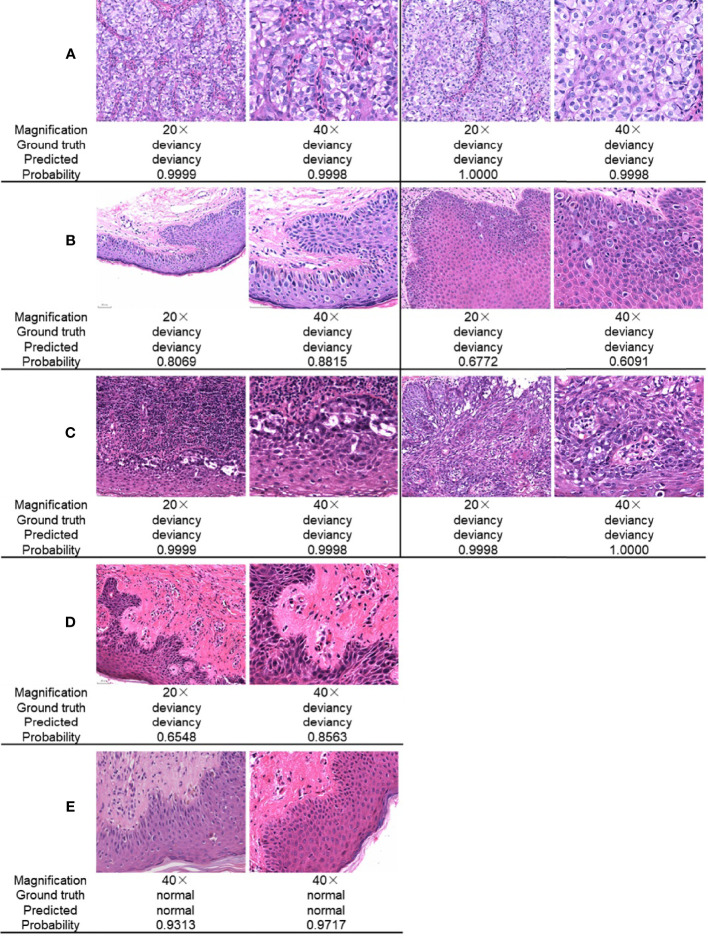
Comparison of ResNet34 prediction results at different magnifications. Paget’s cells are nested distribution **(A)** and diffuse distribution **(B)** with relatively clear vision. **(C)** Paget’s cells are distributed with various types of inflammatory cells. **(D)** Paget’s cells presented atypical morphology in the early stage of the disease. **(E)** Normal skin.

**Figure 5** gives a comparison of the classification results for the skin pathological image at magnifications of ×20 and ×40, respectively. The ground truth, the predicted result, and the corresponding prediction confidence for each image are given. In **Figure 5A**, there are Paget’s cell images with nested distribution. ResNet34 can predict the pathology as deviancy with high probability at different image magnifications. In **Figure 5B**, there are Paget’s cell images with diffuse distribution. ResNet34 can predict the category of the image as deviancy, but the probability of prediction is relatively low, and the probability of predicting pathological images as deviancy at a magnification of ×40 is not significantly different from that at a magnification of ×20. In **Figure 5C**, there is a pathological image of Paget’s cells distributed in more inflammatory cells, which is more difficult to distinguish, and the magnification of the image has less influence on the probability of predicting probability. For pathological images with early lesions shown in **Figure 5D**, ResNet34 can predict its class, but the prediction probability is relatively low and the prediction confidence is more easily affected by magnification. For the normal skin pathological images shown in **Figure 5E**, ResNet34 can predict the category with high probability.

**Figure 6:**
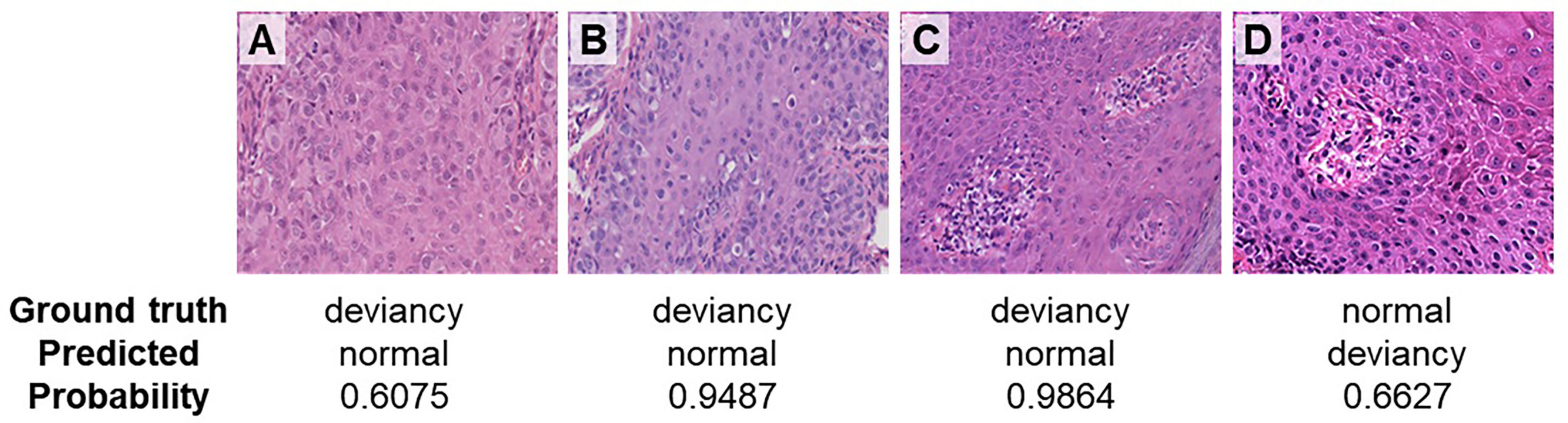
Incorrect prediction results at a magnification of ×40. **(A)** Paget’s cells presented atypical morphology in the early stage of the disease. **(B)** Paget’s cells distributed scattered. **(C)** Paget’s cells are distributed with various types of cells. **(D)** Normal skin image is incorrectly predicted as deviancy.

**Figure 6** gives the incorrect prediction results at a magnification of ×40. **Figures 6A–C** shows cases where the deviancy skin image is incorrectly predicted as a normal category. The possible reason that leads to the incorrect prediction of case A may be due to the relatively large number of cell types, where the cellular atypia of tumor cells was not obvious in the early stage of the disease and the network has more difficulty in identifying the tumor cells within them. In case B, tumor cells distributed more scattered may result in the wrong prediction. In case C, a variety of cell types including vascular endothelial cells, red blood cells, etc. make the network difficult to identify tumor cells, leading to the incorrect prediction. **Figure 6D** is the case where the normal skin image is incorrectly predicted as deviancy, probably because there are several cell types concentrated around the blood vessel and with somewhat heterogeneous nuclei. Therefore, even at high magnification, the relatively large number of cell types and distribution morphology of tumor cells in pathological images can lead to the occurrence of model prediction errors.

**Figure 7:**
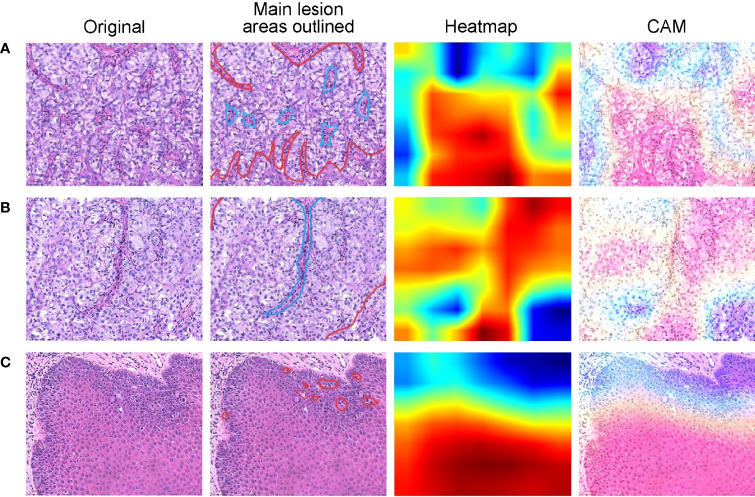
Heatmaps of pathological images show regions of interest identified by the deep learning models. Representative cases of Paget’s cells are distributed with various types of cells **(A)**, arranged in nests **(B)**, or dispersedly **(C)**. For each case, original skin pathological images are listed in the first column. In the second column, the boundaries of tumor and normal tissues are outlined by red and blue lines, respectively. The third column shows the CAMs superimposed on original images and the fourth column shows their corresponding heatmaps.

We use the class activation map (CAM) (21) to visualize the regions of interest when the network model is performing a classification task. CAM is the weighted linear sum of the presence of visual patterns at different spatial locations. By simply upsampling the class activation map to the size of the input image, we can identify the regions most relevant to the particular category. Three representative examples are shown in **Figure 7**, where original skin pathological images are listed in the first column. In the second column, main lesion areas are outlined by the pathologist in the skin pathology image. In this column, boundaries of tumor and normal tissues are outlined by red and blue lines, respectively. The third column shows the CAMs superimposed on original images and the fourth column shows their corresponding heatmaps. The warmer color in the heatmap indicates that the network pays more attention to the part of the feature, which has a greater impact on the classification result. When Paget’s cells are nested (**Figure 7A**), the color distribution of the heatmap shows that the network does pay more attention to the lesion area when perform prediction, and therefore this area has a greater influence on the category determination. When Paget’s cells are diffusely distributed (**Figure 7C**), the color distribution in the heatmap does not give a good indication of the area.

## 4 Conclusion

Deep learning solutions have shown great potential in the field of digital pathological diagnosis. Previous studies mainly focused on melanoma, BCC, and SCC, which constitute 98% of all skin cancers. However, there are some rare skin cancers, including EMPD, that are ignored by most algorithms. Compared with existing works, we for the first time used the deep learning method in Asian EMPD pathological image rapid screening to distinguish between Paget’s cells and normal cells by a retrospective single-center study. Our study provided a prediction model with high accuracy (0.93548 at ×20 magnification and 0.95522 at ×40 magnification) and Auc-score (0.95238 at ×20 magnification and 0.95235 at ×40 magnification), which drew repeatable results of pathological image analysis within a few milliseconds. It is quantitative, fast, and repeatable to empower grassroots pathologists’ efficiency and accuracy as well as to ultimately provide better patient care.

Proportion of skin cancer in overall cancer represents 4%–5% in Hispanics, 2%–4% in Asians, and 1%–2% in Blacks, as reported respectively (22). Deep learning networks validated for the diagnosis of skin cancer in fair-skinned people has a greater risk of misdiagnosing those with darker skin (23). Han et al. trained a deep learning algorithm by using a dataset composed of skin lesions from Asians and found that the diagnostic accuracy was 81%. However, when the same algorithm was applied to the Caucasian database, the accuracy fell to 56% significantly, indicating that persons of a different race, ethnicity, or skin type might influence the diagnostic accuracy (24). Therefore, AI diagnostic systems need to be trained with more datasets of different types of skin cancer as well as different skin types to further improve accuracy among people of all races and colors. In the future, we will further expand our sample size by conducting multicenter prospective research, explore more ways like combining our dataset acquired by other modalities, such as dermoscopic images, and optimize the training process to simulate the actual clinical diagnosis process by multimodal learning.

## Data Availability Statement

The raw data supporting the conclusions of this article will be made available by the authors, without undue reservation.

## Ethics Statement

The studies involving human participants were reviewed and approved by the Huashan Institutional Review Board (HIRB) (ethical review approval number: 2021-901). The patients/participants provided their written informed consent to participate in this study.

## Author Contributions

HW and HC designed the experiments. XW reviewed the EMPD cases. ZY wrote the code. LY and ZS performed the experiments and analyzed the results. JX, BD, and SZ conceived the project. All authors contributed to preparation of the manuscript. All authors contributed to the article and approved the submitted version.

## Funding

This work was supported by the National Natural Science Foundation of China (81703112), Shanghai Municipal Health Commission Science Foundation (20174Y0235), Natural Science Foundation of Jiangsu Province (BK20200745), Natural Science Foundation for Colleges and Universities of Jiangsu Province (20KJB510022), and Medical Engineering Fund of Fudan University (yg2021-032).

## Conflict of Interest

The authors declare that the research was conducted in the absence of any commercial or financial relationships that could be construed as a potential conflict of interest.

## Publisher’s Note

All claims expressed in this article are solely those of the authors and do not necessarily represent those of their affiliated organizations, or those of the publisher, the editors and the reviewers. Any product that may be evaluated in this article, or claim that may be made by its manufacturer, is not guaranteed or endorsed by the publisher.
